# Expert tumor annotations and radiomics for locally advanced breast cancer in DCE-MRI for ACRIN 6657/I-SPY1

**DOI:** 10.1038/s41597-022-01555-4

**Published:** 2022-07-23

**Authors:** Rhea Chitalia, Sarthak Pati, Megh Bhalerao, Siddhesh Pravin Thakur, Nariman Jahani, Vivian Belenky, Elizabeth S. McDonald, Jessica Gibbs, David C. Newitt, Nola M. Hylton, Despina Kontos, Spyridon Bakas

**Affiliations:** 1 Center for Biomedical Image Computing and Analytics (CBICA), University of Pennsylvania, Philadelphia, PA 19104 USA; 2 Department of Radiology, Perelman School of Medicine, University of Pennsylvania, Philadelphia, PA 19104 USA; 3 Department of Pathology and Laboratory Medicine, Perelman School of Medicine, University of Pennsylvania, Philadelphia, PA 19104 USA; 4 University of California San Francisco (UCSF), San Francisco, CA 94115 USA

**Keywords:** Computer science, Breast cancer, Research data, Biomedical engineering, Scientific data

## Abstract

Breast cancer is one of the most pervasive forms of cancer and its inherent intra- and inter-tumor heterogeneity contributes towards its poor prognosis. Multiple studies have reported results from either private institutional data or publicly available datasets. However, current public datasets are limited in terms of having consistency in: a) data quality, b) quality of expert annotation of pathology, and c) availability of baseline results from computational algorithms. To address these limitations, here we propose the enhancement of the I-SPY1 data collection, with uniformly curated data, tumor annotations, and quantitative imaging features. Specifically, the proposed dataset includes a) uniformly processed scans that are harmonized to match intensity and spatial characteristics, facilitating immediate use in computational studies, b) computationally-generated and manually-revised expert annotations of tumor regions, as well as c) a comprehensive set of quantitative imaging (also known as radiomic) features corresponding to the tumor regions. This collection describes our contribution towards repeatable, reproducible, and comparative quantitative studies leading to new predictive, prognostic, and diagnostic assessments.

## Background & Summary

The spatial manifestation of inter- and intra-tumor heterogeneity in breast cancer is well established^1,2^. Current breast cancer diagnosis and subsequent disease management primarily occurs on the basis of histopathologic assessment and biomarkers, which are derived from the sampled tissue. Utilization of biopsies and conventional biomarkers cannot fully capture the intra-tumor heterogeneity, as they are limited by the tissue sampling error, leading to over- or under-treatment. As such, there is a clinical need to characterize the intra-tumor heterogeneity to better understand this disease and its progression mechanisms.

The use of magnetic resonance imaging (MRI) in breast cancer screening, diagnosis, and treatment management, allows for the non-invasive and longitudinal sampling of disease burden^3,4^. Beyond the conventional and qualitative uses of MRI in breast cancer disease management, the field of radiomics, broadly defined as the extraction of high-throughput visual and sub-visual cues derived from medical imaging^5–7^, has allowed for a quantitative characterization and assessment of the breast tumor disease burden. This has led to the development of prognostic and predictive radiomic biomarkers that capture breast intra-tumor heterogeneity, promoting personalized clinical decision making^8^.

Clinical and computational studies analyzing the radiologic presentations of breast tumor disease burden require ample and diverse data to ensure robust characterization. Publicly available datasets, such as those hosted through The Cancer Imaging Archive (TCIA www.cancerimagingarchive.net)^9^, created by the National Cancer Institute (NCI) of the National Institutes of Health (NIH), provide large study cohorts for meaningful research development. Furthermore, such datasets^10–13^ allow for study reproducibility and analyses comparisons across varying institutions, promoting increasingly robust conclusions. However, publicly available radiographic scans require accompanying expertly annotated ground truth tumor annotations to ensure accurate study comparisons and reproducible analyses. Furthermore, any computational analyses, including radiomics-based pipelines, require standardized image normalization and feature parameter selections for consistent analyses^6,7,14–16^.

To address this limitation, this manuscript provides the ‘I-SPY1-Tumor-SEG-Radiomics’ collection, which extends the current TCIA collection ‘I-SPY1’ (https://wiki.cancerimagingarchive.net/display/Public/I-SPY1)^17,18^, with segmentations labels and radiomic features panel for the ACRIN 6657/I-SPY1 TRIAL cohort. The latter contains dynamic contrast enhanced (DCE) MRI images of women diagnosed with locally advanced breast cancer who underwent longitudinal neoadjuvant chemotherapy^17,18^. The primary goal is to allow standardized expert image annotations and radiomic features for researchers to conduct reproducible analyses. To this end, annotations and radiomic features for the baseline (pre-treatment) images of *n* = 163 women have been provided. Based on the analyses that needs to be performed, the selected cohort includes women with baseline (T1) DCE-MRI with at least two post-contrast images for future studies wishing to explore dynamic assessments of breast tumor behavior and treatment response prediction. For each patient visit, three MRI scans are provided over the duration of a single contrast administration: a pre-contrast image, and two post-contrast images. All provided images are pre-operative and pre-treatment. Two sets of annotated labels are provided: i) structural tumor volume (STV) segmentations assessed by an expert board-certified breast radiologist, and ii) functional tumor volume (FTV) segmentations, as described in prior studies^18,19^. While FTV segmentations can provide an assessment of tumor vascularity and perfusion, they are limited in describing the entire structural tumor burden as they only account for voxels of a region of interest (ROI) above a specific intensity threshold. In contrast, the provided STV segmentations annotate the entire structural region (i.e., the whole extent) of the primary lesion. The STV segmentations have been used in prior studies in which radiomic features extracted from the STV region resulted in improved prognostic performance than FTV values^20^. Preliminary evaluation of radiomic features extracted from STV defined primary lesion volumes has demonstrated improved prognostic performance over established clinical covariates^21^.

Additionally, the data cohort includes a comprehensive panel of radiomic features characterizing breast tumor morphology, intensity, and texture. This panel of radiomic features is extracted in compliance with the Image Biomarker Standardization initiative (IBSI)^7^, using the publicly available Cancer Imaging Phenomics Toolkit (CaPTk,https://www.cbica.upenn.edu/captk)^22–24^.

The availability of annotations characterizing the functional active regions around the lesion’s ROI, the entire primary lesion structure, and the computed radiomic features can enable for the development of prognostic and predictive biomarkers characterizing breast tumor heterogeneity through the direct utilization of the TCIA ACRIN 6657/I-SPY1 TRIAL data potential in clinical and computational studies, but importantly can contribute to repeatable, reproducible, and comparative quantitative studies enabling direct utilization of the TCIA I-SPY collection.

## Methods

### Data collection

**Fig. 1:**
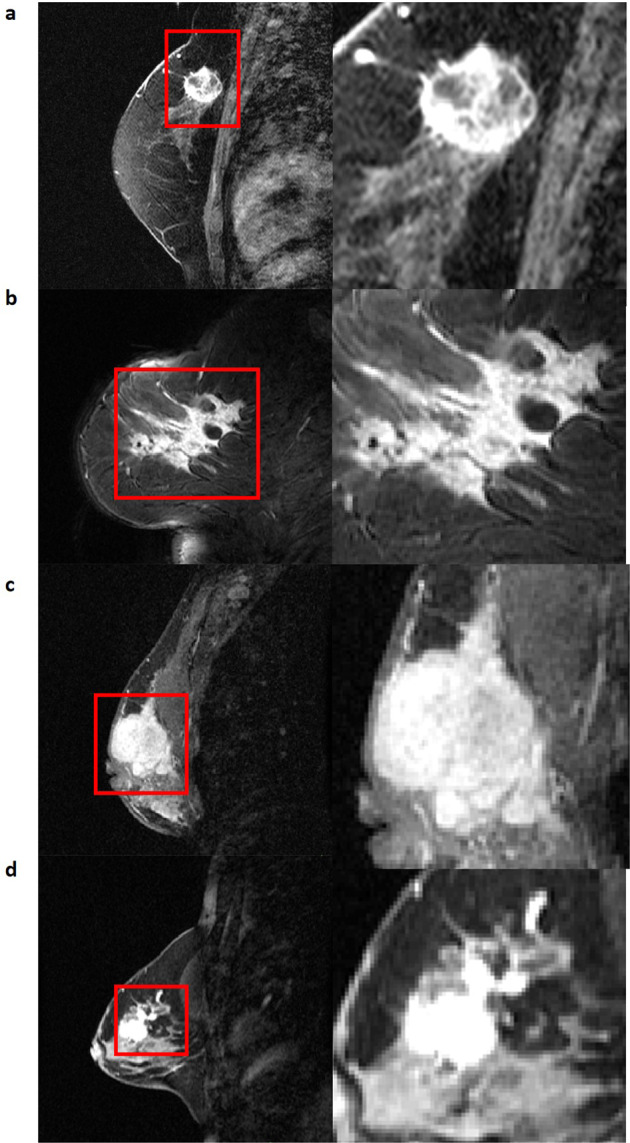
Four representative breast tumors demonstrating spatial intratumor heterogeneity.

The ACRIN 6657/I-SPY1 TRIAL^17,18^ enrolled *n* = 237 women with their consent from May 2002 to March 2006. From this cohort, *n* = 230 women met the eligibility criteria of being diagnosed with locally advanced breast cancer with primary tumors of stage T3 measuring at least 3 *cm* in diameter^18^. The pre-operative DCE-MRI images of 222 women were publicly available via The Cancer Imaging Archive (TCIA)^9^. From this TCIA set, 15 women were excluded for our present study, due to incomplete DCE acquisition scans. A subsequent 44 women were also excluded due to either incomplete histopathologic data or recurrence free survival (RFS) outcome, or missing pre-treatment DCE-MRI scans. This resulted in the inclusion of *n* = 163 women for this study, for whom at least two post-contrast scans from the baseline pre-treatment DCE-MRI scans were available. Women underwent neoadjuvant chemotherapy with an anthracycline-cyclophosphamide regimen alone or followed by taxane. All women underwent longitudinal DCE-MRI imaging on a 1.5 T field-strength system. Distributions of patient histopathologic characteristics and image scanner manufacturer details can be found in Tables 1 and 2. An exemplary illustration showing the spatial intratumor heterogeneity is shown in Fig. 1. The complete clinical metadata is available in the Supplementary Table.

**Table 1:** Summary of patient histopathologic characteristics from study cohort.

Selected patient characteristics	Cases without future recurrent event 119 (73% of total cases)	Cases with future recurrent event: 44 (27% of total cases)
Age (Min., Max., Median)	27.9, 68.8, 48.8	28.8, 68.3, 48.8
Hormone receptor positive	67 (56%)	25 (56%)
HER2 positive	34 (29%)	18 (40%)
Triple Negative	29 (24%)	10 (23%)

**Table 2:** Scanner manufacturer and model name for study cohort.

Manufacturer	Model Name	Number of Cases	Percentage
GE Medical Systems	Genesis Signa	95	58%
	Signa Excite	16	10%
Philips	Intera	10	6%
	Gyroscan Intera	2	1%
Siemens	Magnetom Vision	15	9%
	Magnetom Vision Plus	4	3%
	Sonata	21	13%

### Preprocessing

The preprocessing procedures involved in preparing the data for further analyses were conducted using the Cancer Imaging Phenomics Toolkit (CaPTk)^22–24^, and they are outlined as follows:

1. **Image format conversion:** For each patient, baseline images were converted to the Neuroimaging Informatics Technology Initiative (NIfTI)^25^ file format from the publicly available DICOM scans. This format does not include any identifiable information as the DICOM headers hold, and only preserves the actual imaging information and the necessary information to define the data in the physical coordinates.
2. **Bias Field Correction:** All the converted NIfTI images were bias corrected to rectify any non-uniformity associated with the magnetic field of the MRI scanner^26,27^.
3. **Data harmonization:** This step is required to ensure consistency in the entire dataset as described below.
  - **Resampling:** The raw I-SPY images have different voxel resolutions, preventing cohesive analysis across the entire dataset. To mitigate this, all the images were resampled to the standard 1*mm*^3^ isotropic resolution to ensure harmonized processing for computational algorithms. This resolution is chosen because this resizes all the images to a size which can fit in the GPU memory (more details will be explained later)
  - **Z-Scoring:** After the images are resampled, we Z-score the images using instance level (considering all timepoints of the given patient rather than entire dataset) statistics of mean and variance. Z-scoring is a widely accepted method from extended observations^28–31^, that normalizing every single multi-timepoint scan (i.e., instance-level normalization) to zero mean and a unit variance helps to improve algorithmic generalizability and to preserve the relative intensity differences between the pre- and post-contrast excitation scans.

### DCE-MRI NIfTI volumes

Three volumes have been provided for each patient from the pre-operative, pre-treatment visit. These images include the pre-contrast administration MRI scan (0000), first post-contrast image (0001), and second post-contrast image (0002).

### Expert tumor annotations

From the NIfTI images, the functional tumor volume (FTV) segmentation was identified within the region of interest (ROI), provided through TCIA, from the signal enhancement ratio image, as previously described^18,32^. In order to generate the structural tumor volume (STV) segmentations, voxels outside of the largest contiguous volume region and voxels greater than 2 *cm* away from the largest contiguous volume region, within the FTV, were manually removed. Our expert board-certified breast radiologist then identified the primary lesions in each of the *n* = 163 baseline DCE-MRI images using the manually cleaned, FTV segmentation as a guide. The first-post contrast image for each case was used by the radiologist to delineate the entire 3-D primary tumor segmentation for each patient. Satellite lesions were not considered in the primary tumor segmentations. ITK-SNAP (www.itksnap.org)^33^ was utilized to perform the manual delineations.

### Computationally-generated annotations

A 3D Convolutional Neural Network based on U-Net^34^, with residual connections^35^, was trained on all the preprocessed 3 timepoints to perform automated segmentations of the STV and the code has been made available for reproducibility. The models are trained using the Multi-class *Dice*^36^ Loss function^37^ with on-the-fly data augmentation techniques such as ghosting, blur, and gaussian noise applied in a random manner with a given probability for each type of augmentation^38^. All the experiments are done using *nested k-fold* cross validation and the median *Dice* score across the holdout folds is 0.74. An initial learning rate of 0.01 is used, which is varied in a linear triangular fashion having a minimum learning rate of 10^−3^ times the initial learning rate. We use the Stochastic Gradient Descent optimizer to update weights of our network.

### Radiomic features

An comprehensive array of 370 unique features were extracted. These are from 8 different feature families, based on intensity statistics (n = 20), morphology (n = 21), histograms (n = 285), Gray-level co-occurrence matrix (GLCM) (n = 8), Gray-level run-length matrix (GLRLM) (n = 12), Gray-level size zone matrix (GLSZM) (n = 18), Neighborhood gray tone difference matrix (NGTDM) (n = 5), and Local binary patters (LBP) (n = 1). We used non-filtered images after the first post-contract injection that were bias-corrected, resampled and z-score normalized. The radiomic features were then extracted from the region defined by the STV. The extraction was done using the Cancer imaging Phenomics Toolkit (CaPTk, www.cbica.upenn.edu/captk)^22–24^. CaPTk is an open-source software toolkit, which offers functionalities to extract a wide array of radiomic features compliant with the image biomarker standardisation initiative (IBSI)^7^, the Quantitative Imaging Network^6^, and has been extensively used in radiomic analysis studies^39–43^. The exact parameters used for the radiomic analysis are available through TCIA’s repository, at 10.7937/TCIA.XC7A-QT20^44^.

## Data Records

We are using the data^17^ published through the ACRIN 6657/I-SPY1 TRIAL study^18^. Specifically, we selected baseline subjects for whom at least two pre-operative post-contrast scans were available. The raw and generated data, which includes the preprocessed images in isotropic resolution of 1*mm*^3^, the expert and computationally-generated annotations, and the extracted radiomic features, have been made available through TCIA’s Analysis Results Directory www.cancerimagingarchive.net/tcia-analysis-results/ using 10.7937/TCIA.XC7A-QT20^44^. The computationally generated annotations can stand as a benchmark for improving segmentation algorithms related to this data in future computational studies.

## Technical Validation

### Data collection

**Fig. 2:**
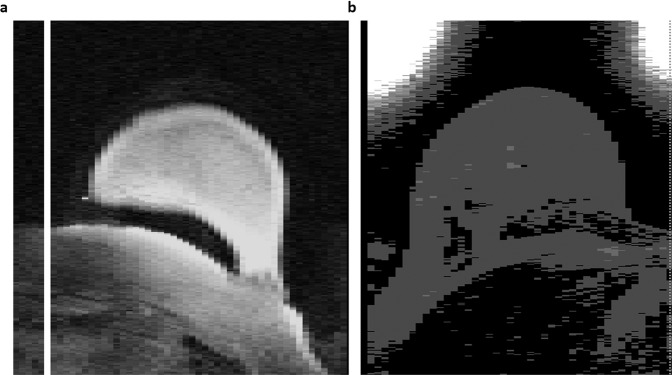
Representative image slice where image artifact is present. (**a**) Visualization of image artifact for case 1183, (**b**) visualization of image artifact for case 1187. These image artifacts do not affect the intensity values within anatomical breast region.

The dataset was directly downloaded from TCIA and quantitatively analyzed to ensure all images have a defined coordinate system and contain non-zero pixel values. Two cases, 1183 and 1187, had white image artifacts outside of the breast region. While these artifacts do not affect intensity distributions within the anatomical breast or the corresponding lesion segmentations, they may cause difficulties in image visualization, and downstream analyses. These artifacts were present in images directly downloaded from TCIA (illustrated in Fig. 2). Additionally, qualitative assessment was performed to look for any visual data corruption.

### Preprocessing

Each step of preprocessing was followed by manual qualitative assessment of the image to ensure data validity. In addition, quantitative assessment was performed following the data harmonization step to ensure that the entire dataset had the same parametric definition (i.e., same resolution and pixel intensity distribution).

### Expert tumor annotations

The expert annotated STV segmentations were qualitatively assessed, manually edited and approved by a board certified, fellowship-trained breast radiologist.

### Computationally-generated annotations

**Fig. 3:**
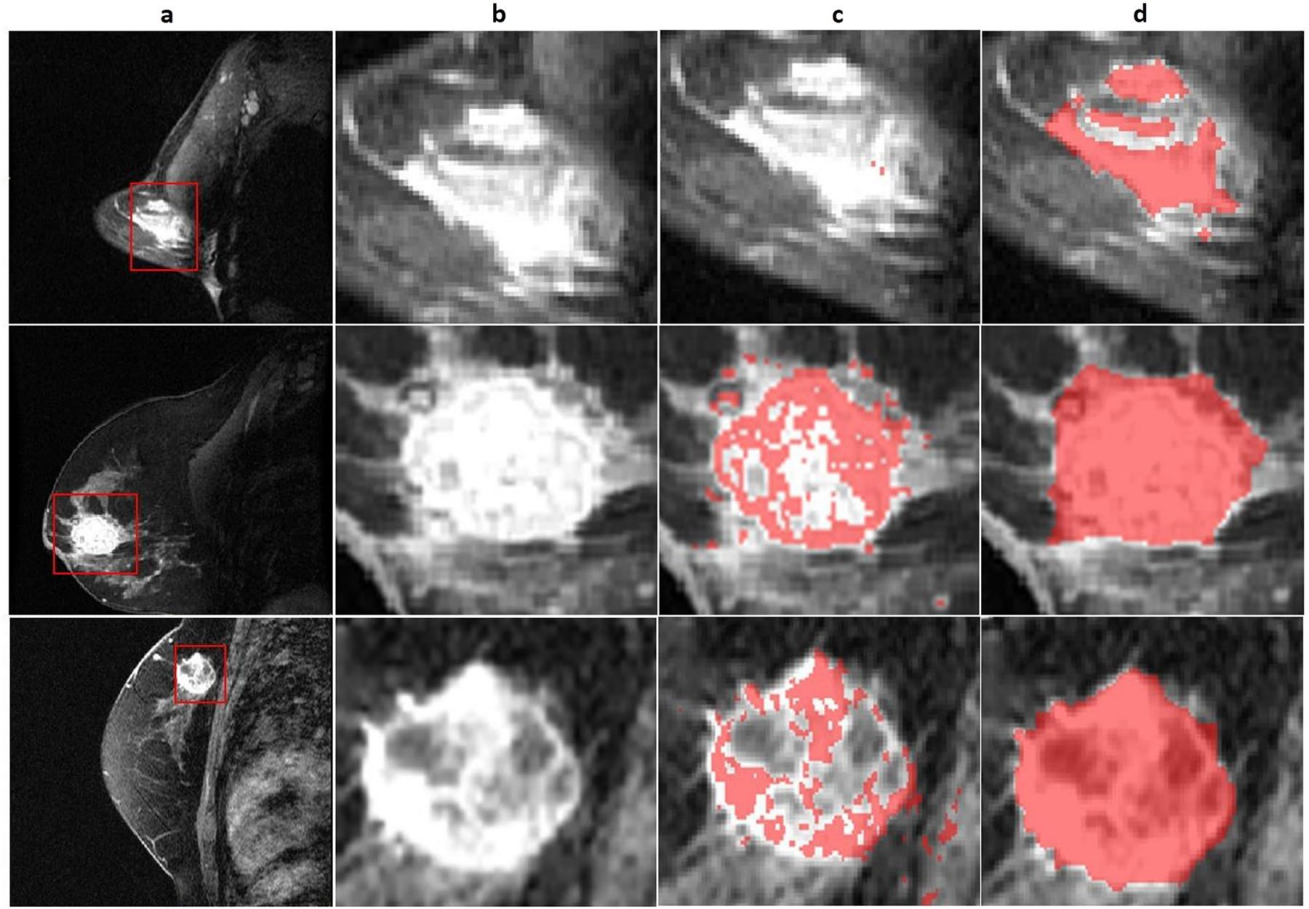
Three representative single slice tumor segmentations. (**a**) First-post contrast image of entire breast. (**b**) Primary tumor region of interest. (**c**) Functional tumor volume (FTV) segmentation (**d**) Structural tumor volume (STV) segmentations which have been expert annotated. Rows showcase different representative images for each case.

The FTV annotations were quantitatively compared with the corresponding STV annotations using the *Dice* score in order to quantify the difference between the two annotations. Additionally, a qualitative analysis was performed for the best and worst performing cases (illustrated in Fig. 3).

### Feature extraction

Considering the mathematical formulation of these features, it is possible for a division by zero to occur (lack of heterogeneity or very small number of voxels). In CaPTk, we provide “not a number” for the result of these features to provide a position of clarity for the user to make subsequent downstream analyses more coherent based on the entire population. We acknowledge this could be provided as “inf” instead, but we are providing this as “NaN” to have parity between various programming languages and processing protocols.

## Usage Notes

This collection of images (both normalized and resampled) and accompanying annotations can be analyzed using different tools or software. We provide all the annotations in a research-friendly NIfTI format to allow users to read the images and annotations through many programming languages such as C++, Python, R, or others. The data is accompanied by a XSLX file that provides additional information about each subject.

## Supplementary information

Supplementary Table - Original clinical metadata

## Data Availability

In favor of transparency and reproducibility, but also in line with the scientific data principles of Findability, Accessibility, Interoperability, and Reusability (FAIR)^45^, we have made the tools used to generate the data for this study publicly available^38^. Specifically, the CaPTk platform^22–24^, version 1.8.1, was used for all the preprocessing steps. CaPTk’s source code and binary executables are publicly available for multiple operative systems through its official GitHub repository (https://github.com/CBICA/CaPTk). The implementation and configuration of the U-Net with residual connections, used in this study, can be found in the GitHub page of the Generally Nuanced Deep Learning Framework (GaNDLF), version 0.0.14 (https://github.com/CBICA/GaNDLF). Finally, ITK-SNAP^33^, was used for all the manual annotation refinements.
